# Use of acetaminophen in relation to the occurrence of cancer: a review of epidemiologic studies

**DOI:** 10.1007/s10552-016-0818-2

**Published:** 2016-11-10

**Authors:** Noel S. Weiss

**Affiliations:** University of Washington, Seattle, WA USA

**Keywords:** Acetaminophen, Cancer epidemiology, Cancer incidence

## Abstract

Acetaminophen has several pharmacologic properties that suggest it could be carcinogenic in human beings. A number of epidemiologic studies have been conducted to examine whether use of acetaminophen actually predisposes to the occurrence of one or more forms of cancer. There are inherent limitations to many of these studies, including the inaccurate identification of users and nonusers of acetaminophen, relatively short follow-up for cancer incidence, and the potential for confounding by indication. The present manuscript reviews the results of epidemiologic studies of acetaminophen use in relation to cancer incidence published through the end of 2015. The limitations of the underlying studies notwithstanding, some interim conclusions can be reached. For all but several forms of cancer, there is no suggestion that persons who have taken acetaminophen are at altered risk, even persons who have consumed a large quantity of the drug or those who have taken it for an extended duration. While in some studies the incidence of renal cell carcinoma has been observed to be increased among acetaminophen users, several other studies have failed to observe any such association; the reason for the discrepant findings is unclear. Some of the small number of studies that have presented data on the incidence of lymphoma, leukemia, and plasma cell disorders have found the risk to be modestly higher in users than nonusers of acetaminophen, but the results of other studies of these malignancies will be needed to gauge the possible role of publication bias as the basis for the positive results.

## Introduction

Acetaminophen (AC) is a constituent of many analgesic preparations. AC is a metabolite of the drug phenacetin, the use of which is associated with an increased risk of tumors of the bladder and renal pelvis [1]. In experimental systems AC administration in supra-therapeutic doses leads to inhibition of DNA repair, and some of the metabolites of AC have been found to be mutagenic [2, 3].

Because of concerns regarding carcinogenicity, a number of epidemiologic studies have examined the possibility that use of AC can influence the occurrence of cancer in human beings. The present review seeks to summarize the results of those studies of this issue that were published through the end of 2015.

## Methods

The starting point of this review was a list of studies of AC use and cancer incidence that was assembled in 2011 by the California Office of Environmental Health Hazard Assessment. This was augmented by searches of PubMed for more recent articles, using the search terms acetaminophen, paracetamol (another name for AC), and neoplasms.

An attempt was made to estimate the relative risk (RR) of a given cancer in relation to use of AC from each of the studies. In cohort studies, in which the occurrence of various forms of cancer is compared between users and nonusers of AC, it is possible to directly calculate the RR. A value of the RR above one suggests an increased incidence in AC users; a value of the RR below one suggests a decreased incidence. From case–control studies, in which a comparison is made between a history of AC use in persons with a given form of cancer (cases) and in other persons (controls), the ratio of the odds of exposure (OR) between cases and controls serves as a good approximation of the RR. For both the RR and the OR, the degree of statistical uncertainty is provided by the 95% confidence interval (CI); this indicates the range of values that is statistically compatible with the data observed in a particular study.

In describing the results of individual cohort and case–control studies, emphasis was given to the data presented regarding relatively more frequent and/or relatively more long-term use of AC. The basis for this choice is the expectation that if any altered risk of a given form of cancer is associated with prior AC use, that association would be most prominent in persons who had heavy or prolonged exposure.

Because of the heterogeneity of the known causes of cancers of different organs, the results are presented separately for each of the individual forms of cancer for which data are available.

## Results

### Esophageal adenocarcinoma

Two case–control studies, one in Northern Ireland and the other in Australia, examined the potential relation of use of AC to the incidence of esophageal adenocarcinoma. Neither found evidence of an association. In Northern Ireland [4], the OR associated with ever use of AC (at least once per week for six months or more, ignoring use in the year prior to diagnosis) was 0.8 (95% CI 0.5–1.5). In the Australian study [5], the OR associated with at least weekly use was 0.9 (95% CI 0.5–1.4).

### Esophageal squamous cell cancer

The only data pertaining to esophageal cancers of this type come from the Australian study [5]. While a modest positive association was seen for at least weekly use (OR 1.3), this result was well within the limits of chance given no true association (95% CI 0.7–2.3).

### Cancers of the colon and rectum

Most studies of the occurrence of colorectal cancer in users of AC did not separately examine colon and rectal cancers. None of these studies observed any hint of an association with AC use.

- In a UK cohort study during 1994–1997 [6], the relative risk among present AC users of at least one year’s duration was 0.9 that of nonusers (95% CI 0.7–1.1), and the corresponding RR among past AC users of at least one year’s duration was 0.9 (95% CI 0.5–1.4)
- In a hospital-based case–control study in Ohio [7], the OR associated with AC use of at least 1 day per week for at least one year was 0.8 (95% CI 0.4–1.6)
- A UK case–control study [8] observed an OR of 1.0 associated with a history of 30 or more prescriptions for AC (95% CI 0.7–1.4)
- In a US hospital-based case–control study [9], for men the OR associated with daily AC use for one or more years was 1.1 (95% CI 0.4–3.2). For women, the corresponding OR was 0.6 (95% CI 0.3–1.3)
- A case–control study in Georgia [10] stated that no altered risk of colorectal cancer was associated with use of AC, but no quantitative estimate was provided
- A US cohort study [11] found the risk of colorectal cancer in persons who had taken AC at least four days per week for at least 4 of the prior 10 years to be 0.8 times that of AC nonusers (95% CI 0.5–1.4).

Two studies reported the results for colon and rectal tumors separately. In a Danish cohort study [12], no appreciable association with AC use (and no other analgesic) of at least a year’s duration was observed for either site: OR for colon cancer = 0.9, 95% CI 0.7–1.3; OR for rectal cancer = 1.2, 95% CI 0.8–1.7. In the American Cancer Society cohort study [13], there was no association observed for colon cancer mortality in relation to AC use of at least 16 days in the month prior to questionnaire completion (RR not provided), but the RR for rectal cancer mortality in that study was 3.1 (95% CI 1.1–8.5). Apart from the wide confidence interval, the latter result must be interpreted very cautiously, since the failure of the above six studies to present separate results for colon and rectal cancer could well be due to there not being a difference between the two sub-sites with respect to a lack of association with AC use.

### Pancreatic cancer

Three studies to date have evaluated the occurrence of pancreatic cancer in relation to use of AC, and none found any hint of an increase in risk. The first, a cohort study by Thun et al. [13], did not present data specifically for cancer of the pancreas, but stated that more than occasional use of AC during the prior month was “not associated with any digestive tract cancer.” In the large Danish cohort study [12], 51 cases of pancreatic cancer were diagnosed among persons who had received at least one prescription for AC (after allowing for an induction period of one year), but this was almost exactly that expected among persons who had not received such a prescription (RR 0.9, 95% CI 0.7–1.2). In the clinic-based case–control study of Tan et al. [14], the OR associated with use for more than one day per month of AC use was 1.0 (95% CI 0.8–1.3). The corresponding ORs for use six or more days per week (0.7, 95% CI 0.4–1.6) and three or more times per day (1.1, 95% CI 0.8–1.6) also were not elevated.

### Lung cancer

Four studies assessed the risk of lung cancer among users of AC. The only one to find an appreciable association [12] observed an RR of 1.6 (95% CI 1.4–1.7) but was not able to control for the potentially confounding influence of cigarette smoking. In their cohort study, Walter et al. [11] observed RRs of 1.2 and 1.1 among persons with “low” and “high” cumulative exposure to AC in the 10-year period prior to the start of follow-up (“high” being defined as use for at least four days per week for at least 4 years), after adjustment for pack-years of smoking and a number of other variables. Similarly, the report by Olsen et al. [15], of the collective results of two Danish studies, described a smoking-adjusted RR of 1.1 (no CI provided) associated with AC use during the 1–5 years prior to diagnosis. A small American case–control study [16] observed a smoking-adjusted OR for AC use of 1.4, but the confidence interval around this estimate (0.5–3.4) was wide and easily included the null.

### Breast cancer

To date there have been 13 publications pertaining to breast cancer incidence in relation to use of AC [8, 11, 17–27]. They have been based on both cohort and case–control studies, have come from both Europe and from North America, and have ascertained information on use of AC from computerized pharmacy data and from interviews/questionnaires. The results consistently have been null. Relative risks (or odds ratios) ranged from 0.8 to 1.1, even for those groups of women with the longest durations or highest frequencies of AC use.

### Endometrial cancer

The results of four cohort studies [11, 12, 28, 29] and three case–control studies [30–32] suggest that regular use of AC (typically defined as at least 1–2 tablets per week for at least one month) is not associated with the occurrence of cancer of the endometrium. Relative risks and odds ratios ranged from 0.9 to 1.2 across the studies, with no consistent evidence of an increase in risk with increasing duration of regular use.

### Ovarian cancer

Trabert et al. [33] conducted a pooled analysis of 12 population-based case–control studies of ovarian cancer that obtained medication histories. The overall adjusted OR associated with “regular” use of AC (typically, at least once per week for at least six months) was 1.0 (95% CI 0.9–1.1). This null result is entirely concordant with that observed in cohort studies [12, 28, 34–36] of this question and those relevant case–control studies [37–40] that were not included in the pooled analysis. Also, there was no suggestion of an increased risk of ovarian cancer with long-term use of AC (in those studies that examined duration of use).

### Prostate cancer

When considered as a whole, those studies that were able to examine the occurrence of prostate cancer in relation to relatively longer-term use of AC did not identify any appreciable positive association. Among the cohort studies, Friis et al. [12] observed the number of observed and expected cases among men who had received an AC prescription for at least a year to be identical (RR = 1.0, 95% CI 0.9–1.3). Among men in the American Cancer Society cohort who had taken at least one AC tablet per day for at least five years, mortality from prostate cancer was less than that of male nonusers (RR = 0.6, 95% CI 0.4–0.9) [41]. Walter et al. [11] observed no difference between the incidence of prostate cancer in men who had taken AC (for at least four days per week for at least 4 years) and men who had never regularly taken AC. And Garcia-Rodriguez et al. [42] found an RR of 1.1 associated with current use of AC at the start of follow-up (95% CI 1.0–1.3), a value that did not increase with increasing duration of use. Then, in a case–control study, Salinas et al. [43] observed a similar proportion of cases as controls to have used AC regularly for five or more years (OR 1.2, 95% CI 0.7–1.5).

Several other studies of prostate cancer have reported results regarding AC use [44–47], but these are less useful than the ones cited above due to either their inability to identify longer-term AC users or their very small size.

### Renal cell cancer

A number of studies have examined a possible relation between use of AC and the occurrence of renal cell carcinoma, and the results are mixed. Several case–control studies identified a modest association:

- Among members of Group Health (a prepaid health care plan in Washington state) with cancer of the kidney (the majority of whom would be expected to have had renal cell carcinoma), prior to diagnosis a higher proportion had received two or more prescriptions for AC than had controls [48]. The OR rose steadily with increasing duration of use, from 1.3 for 2–19 prescriptions to 2.6 (95% CI 1.1–6.0) for 40+ prescriptions.
- In a population-based study in Los Angeles [49], regular use of AC prior to the date of diagnosis (not considering the final 2 years) was associated with an OR of 1.7 (95% CI 1.3–2.1). Among persons who typically ingested eight or more grams per week, the OR was 2.1 (95% CI 1.3–3.3).
- In a case–control study within the UK General Practice Research Database [50], a higher proportion of cases than controls had been prescribed AC during the 1–5 years prior to diagnosis. The ORs rose to some extent with an increasing number of prescriptions: 1.4 (95% CI 0.9–2.4 for 1–5, 2.1 (95% CI 0.9–4.6) for 6–19, and 2.3 (95% CI 1.0–5.3) for 20+ prescriptions. The fact that 20 of the 23 cases who had been prescribed AC received their first prescription at least three years prior to cancer diagnosis argues against the hypothesis that symptoms resulting from undiagnosed cancer led to the use of this analgesic.

However, an equal number of case–control studies of renal cell cancer failed to see any appreciable case–control difference in prior AC use:

- Among neither men (OR 0.9, 95% CI 0.4–1.8) nor women (OR 0.6, 95% CI 0.4–1.6) in an Ontario case–control study [51] was a history of use of AC at least every other day for at least one month associated with an increased risk.
- An international study involving four populations [52] observed an OR of 1.1 (95% CI 0.9–1.5) associated with cumulative lifetime AC use of more than 0.1 kg through the years ending 2–4 years prior to diagnosis.
- In a US hospital-based study [53], more than 5 years of regular AC use (i.e., more than twice per week) was not associated with risk of renal cell cancer (OR 1.1, 95% CI 0.5–2.6).

In addition, the results of three cohort studies collectively offer little support for the hypothesis that use of AC can predispose to the occurrence of renal cell cancer:

- In a Danish study [12], the incidence of kidney cancer beginning one year following the receipt of a prescription for AC was 1.3 times higher than expected (95% CI 0.9–1.7) based on the incidence among persons not prescribed AC.
- In the combined populations of the Nurses’ Health Study and Health Professionals Follow-up Study [54], receipt of AC for at least two days per week during the prior 2 years was not associated with an increase in risk, even for use that had been taking place for 4–9 years (RR 1.0, 95% CI 0.6–1.7) or for 10 or more years (RR 1.1, 95% CI 0.7–1.7).
- Among persons enrolled in a cohort study that took place in the Pacific northwest [11], use of AC for at least four days per week for at least 4 years during the 10 years prior to questionnaire completion was not associated with an altered risk during follow-up (RR 1.0, 95% 0.5–2.0).

In summary, there is a suggestion from the results of several studies that persons with a higher than average dose of AC or longer than average duration of use had a modestly increased risk of renal cell cancer. However, the negative results obtained in other studies of this question argue for a cautious interpretation.

### Bladder cancer

Zhang et al. [55] summarized the results of all cohort and case–control studies published prior to November 2012. From these studies, they estimated a relative risk of bladder cancer associated with any regular use of AC to be 1.0 (95% CI 0.9–1.2). Neither was an association observed for higher than average intensity of use (RR 1.1, 95% CI 0.8–1.4) nor for a longer than average duration of use (RR 0.8, 95% CI 0.4–1.5).

### Melanoma

Four cohort studies have examined a possible association between use of AC and melanoma incidence [11, 12, 56, 57], with uniformly null results.

### Other skin cancer

In the three studies of this question, there was no suggestion of an increase in risk. An American case–control study [58] observed that a history of use of AC for seven or more years was associated with a modest decrease in incidence of both squamous cell cancer of the skin (OR 0.7, 95% CI 0.4–1.2) and basal cell carcinoma (OR 0.5, 95% CI 0.3–1.0). In the Nurse’s Health Study, Jeter et al. [56] found that women who used AC 6–7 times each week during the prior two years had nearly the same incidence of squamous cell skin cancer (RR 0.8, 95% CI 0.6–1.0) and basal cell cancer (RR 1.1, 95% CI 1.0–1.2) as AC nonusers. Finally, in the Women’s Health Initiative cohort [59], regular use of AC for five or more years was associated with an RR of 1.1 (95% CI 0.9–1.2) for the aggregate category of nonmelanoma skin cancer.

### Brain cancer

The limited data available do not suggest any increased risk of brain tumors among users of AC. In a Danish cohort study [12], the incidence of brain cancer beginning one year after a person’s first AC prescription was similar to that of other persons (RR 0.8, 95% CI 0.6–1.1). A case–control study [60] observed that use of AC in the 10 years prior to a diagnosis of glioblastoma (minimum of 600 pills) was similar to that of controls during the corresponding period of time (OR 0.9, 95% CI 0.6–1.5). However, the interpretation of the results of the latter study needs to take into account the substantial fraction of cases for whom medication history had been obtained from a surrogate respondent, and the difference in the reported use of AC by type of respondent.

### Lymphohematopoietic neoplasms

#### Lymphoma

The results of three case–control studies of non-Hodgkin’s lymphoma in relation to prior use of AC have been published. In a population-based study in New York state, Kato et al. [61] observed at most a very weak association for daily AC use of at least 6-months duration during the prior 20 years: OR of 1.0 for <3 years of use (95% CI 0.4–2.3), 1.3 for 3–10 years (95% CI 0.5–3.1), and 1.4 for >10 years (95% CI 0.5–4.3). A hospital-based study [62] found that in men, AC use at least once per week for at least six months was associated with an OR of 0.8 (95% CI 0.5–1.2). In women, though the corresponding OR was elevated (1.7, 95% CI 1.2–2.5), there was no further increase with increases in number of pills per day or number of years used. The study of Holly et al. [63] provided no data, but in their manuscript the authors stated that there was no association of non-Hodgkin’s lymphoma with use of AC.

In the cohort study of Walter et al. [64], “high” use of AC (defined as at least four days per week for at least 4 of the past 10 years) was associated with an increased risk of the entity “mature B cell neoplasms other than chronic lymphocytic leukemia, small lymphocytic lymphoma, or plasma cell disorders” (RR 1.8, 95% CI 1.1–2.9). No similar elevation in risk was associated with less intense and/or shorter use (RR 0.8, 95% CI 0.6–1.3).

Becker et al. [65] conducted a case–control study of a heterogeneous group of lymphohematopoietic neoplasms—including non-Hodgkin’s lymphoma, Hodgkin’s lymphoma, and multiple myeloma—in several countries, and observed an OR of 2.3 associated with prior use of AC (95% CI 1.5–3.5). However, no description was provided as to how AC use was defined and/or categorized in this study.

Finally, Chang et al. [66] observed that a higher proportion of patients with Hodgkin’s lymphoma than controls had taken two or more tablets of AC per week in the prior 5 years (OR 1.7, 95% CI 1.3–2.3). The size of the association was unchanged when the analysis was restricted to cases with a lesser degree of symptomatology, arguing against the hypothesis that use of AC had been initiated as a result of the presence of the as-yet-undiagnosed malignancy.

#### Plasma cell malignancies

In a cohort study, Walter et al. [64] observed an increased risk of plasma cell disorders among takers of AC. Persons who had used AC for at least 4 days per week for at least four of the 10 years prior to the baseline questionnaire had a RR of 2.4 (95% CI 1.1–5.4). The RR in persons with a smaller cumulative exposure was 1.6 (95% CI 0.9–3.0). In a hospital-based case–control study of multiple myeloma, Moysich et al. [67] found an even stronger association: The OR associated with “regular” use of AC (at least one pill per week for a period of six months or longer that preceded the current illness) was 3.0 (95% CI 1.7–5.1). The association was somewhat stronger still for daily use (OR 4.4, 95% CI 1.7–11.2) and for a history of use for at least 10 years (OR 3.3, 95% CI 1.5–7.0).

Given that bone pain is an early feature of myeloma/plasma cell disorders, the hypothesis of “reverse causality”—cancer causing analgesic use, rather than the reverse—has to be considered. The results obtained in these two studies suggest that this alternative hypothesis is not the sole explanation: In neither study was the use of analgesics other than AC appreciably associated with risk. And, in both studies, a particularly strong association was present with more than several years of use of AC, use which likely would have started before any cancer symptoms had been present.

#### Leukemia

In a cohort study, Walter et al. [64] examined use of AC in relation to the incidence of myeloid neoplasms (primarily acute myeloid leukemia and myelodysplastic syndrome). Persons who had taken AC for at least four days per week for at least four of the prior 10 years had a risk that was 2.3 times that of nonusers (95% CI 1.2–4.1). In users of AC of lesser intensity or a shorter duration, the RR was 1.6 (95% CI 1.0–2.4). In a hospital-based case–control study of acute leukemia [68], the OR associated with prior regular AC use (defined as at least one tablet per week for six consecutive months) was 1.5 (95% CI 1.0–2.3). However, the size of the association did not rise further in relation to the duration of use or the total number of tablets consumed. The results were similar when cases were restricted to acute myeloid leukemia.

In summary, to date there have been but a very small number of published evaluations of a possible relation between use of AC and the occurrence of plasma cell disorders and of leukemia. Though the results of these generally have suggested an increased risk, the size of the reported increase has not been large. Also, several other studies that have the data to address the question of an altered risk of plasma cell disorders and/or leukemia have not yet weighed in, leaving open the possibility of publication bias. A larger number of studies have reported results for lymphoma, and several of these have observed increases in risk associated with AC use. However, just as often the results for lymphoma have not been positive. At present, while an increased risk of one or more lymphohematopoietic neoplasms among users of AC is a plausible hypothesis, it should not be regarded as anything more than this.

## Discussion

### Barriers to gauging the impact of AC use on cancer risk

Ideally, the potential carcinogenicity of AC would be addressed in a large trial of persons in need of pain relief, in which participants would be assigned at random to receive long-term AC or a placebo and then monitored for cancer incidence for a lengthy period of time. Such a study does not exist. Unfortunately, non-randomized approaches to this question have substantial hurdles to overcome in order for a valid result to emerge:

1. In most populations, AC-containing products are available without a prescription. Thus, cohort studies based on drug prescription data (studies which are commonly employed when gauging the safety of prescription medications) will not identify most AC users. Also, over-the-counter use of AC may be common in a given study population, leading to an attenuation of any true association of cancer incidence with AC use in studies that rely on prescription data alone.
2. Almost all users of AC will suffer from pain, whereas most nonusers will not. If the inflammatory process associated with many types of pain itself is associated with cancer incidence, an observed association between AC use and a given form of cancer could be a spurious one. While it would be possible to compare cancer incidence across groups of users of different analgesics, there would be no clear interpretation of such a comparison, since the possible impact of the other agent(s) on cancer risk could not be evaluated.
3. If a long interval is required for AC to influence the occurrence of cancer, the duration of participant follow-up in cohort studies that rely on prescription records—records that may have only recently become available—may not be adequate to capture the period of altered risk.
4. Some of the case–control studies that have been done employed patients seeking care for noncancerous conditions, or persons seeking cancer screening, as controls. To the extent that the use of AC in such persons does not reflect the use of AC in the underlying population from which the cancer cases were derived, the results will be biased.
5. There is the threat of publication bias: (a) the authors of cohort studies may provide results selectively for those sites of cancer that are observed to be associated with use of AC but not for other sites; and (b) the authors of case–control studies in which a broad range of potential exposures have been examined may provide data on those for which there is an association seen, but not for the other exposures. When conducting a literature review of a possible association between use of AC and cancer risk, both of these potential sources of publication bias could give rise to an exaggeration of any true association (positive or negative).

### Does use of AC predispose to one or more forms of cancer?

Ultimately, the answer to this question must come from the experience of human beings as documented in epidemiologic studies. The results of those studies to date, the limitations listed above notwithstanding, provide reassurance that for the large majority of cancers, there is no increased risk associated with AC use, even when such use is prolonged. However, for several forms of cancer—namely plasma cell disorders and leukemia—there is a suggestion of a higher incidence following use of AC, but this suggestion is based on the results of but a very limited number of studies that have chosen to report results for these conditions. Once data from all relevant studies become available, especially from those with an adequately long duration of follow-up of study participants, we should have a better basis for forming a conclusion regarding a possible connection with use of AC than we do at present.

The results of several epidemiologic studies suggest that the incidence of renal cell carcinoma is increased among users of AC. But, as described earlier, equally numerous and equally well-done studies have observed no alteration in risk. Furthermore, the expectation from studies of increased cancer incidence among users of phenacetin—the drug whose primary metabolite is AC—was that use of AC would be associated with an increased risk of transitional cell tumors of the bladder and renal pelvis, which is not what has been observed. In short, the discrepant results from epidemiologic studies of renal cell carcinoma among users of AC have no ready explanation, and it is unclear if the conduct of additional studies will generate any clearer interpretation.
